# Loss of the Transcriptional Repressor PAG-3/Gfi-1 Results in Enhanced Neurosecretion that is Dependent on the Dense-Core Vesicle Membrane Protein IDA-1/IA-2

**DOI:** 10.1371/journal.pgen.1000447

**Published:** 2009-04-03

**Authors:** Tao Cai, Hiroki Hirai, Tetsunari Fukushige, Ping Yu, Guofeng Zhang, Abner L. Notkins, Michael Krause

**Affiliations:** 1 Experimental Medicine Section, Oral Infection and Immunity Branch, National Institute of Dental and Craniofacial Research (NIDCR), National Institutes of Health, Bethesda, Maryland, United States of America; 2 Section of Developmental Biology, Laboratory of Molecular Biology, National Institute of Diabetes and Digestive and Kidney Diseases (NIDDK), National Institutes of Health, Bethesda, Maryland, United States of America; 3 Protein-Nucleic Acid Interactions Section, Structural Biophysics Laboratory, National Cancer Institute (NCI)-Frederick, Frederick, Maryland, United States of America; 4 Laboratory of Bioengineering and Physical Science, National Institute of Biomedical Imaging and Bioengineering (NIBIB), National Institutes of Health, Bethesda, Maryland, United States of America; Columbia University, United States of America

## Abstract

It is generally accepted that neuroendocrine cells regulate dense core vesicle (DCV) biogenesis and cargo packaging in response to secretory demands, although the molecular mechanisms of this process are poorly understood. One factor that has previously been implicated in DCV regulation is IA-2, a catalytically inactive protein phosphatase present in DCV membranes. Our ability to directly visualize a functional, GFP-tagged version of an IA-2 homolog in live *Caenorhabditis elegans* animals has allowed us to capitalize on the genetics of the system to screen for mutations that disrupt DCV regulation. We found that loss of activity in the transcription factor PAG-3/Gfi-1, which functions as a repressor in many systems, results in a dramatic up-regulation of IDA-1/IA-2 and other DCV proteins. The up-regulation of DCV components was accompanied by an increase in presynaptic DCV numbers and resulted in phenotypes consistent with increased neuroendocrine secretion. Double mutant combinations revealed that these PAG-3 mutant phenotypes were dependent on wild type IDA-1 function. Our results support a model in which IDA-1/IA-2 is a critical element in DCV regulation and reveal a novel genetic link to PAG-3-mediated transcriptional regulation. To our knowledge, this is the first mutation identified that results in increased neurosecretion, a phenotype that has clinical implications for DCV-mediated secretory disorders.

## Author Summary

Within secretory cells, hormones are packaged into vesicles (called DCVs) that are released upon stimulation. The number of DCVs is regulated to meet the secretory demands of the cell by a mechanism that is poorly understood, although a protein in the membrane of DCVs, called IA-2, is thought to play a role. A genetic screen in the nematode *C. elegans* is used, here, to find mutations that mis-regulate the corresponding worm protein called IDA-1. Capitalizing on the simple neuroanatomy of the nematode and its transparency, we visualize IDA-1 protein levels directly in the animal using a fluorescent tag. We find that mutations in the transcription factor PAG-3/Gfi-1 result in elevated levels of IDA-1 protein, increased numbers of presynaptic DCVs, and behaviors consistent with increased neurosecretion. Our results demonstrate that IDA-1/IA-2 protein levels correlate with the biogenesis, utilization, or stability of DCVs. We propose that PAG-3 normally down regulates the production of IDA-1, thus serving as part of the mechanism underlying DCV regulation. This is the first reported mutation that increases DCV numbers and secretion, offering insight into DCV homeostasis and a potential therapeutic target for diseases that would benefit from a boost in neuroendocrine secretion.

## Introduction

Dense core vesicles (DCVs) mediate the storage and secretion of hormones (e.g. insulin) and neuropeptides (e.g. vasoactive intestinal peptide, VIP) from a variety of neuroendocrine cells (e.g. pancreatic β-cells). The biogenesis of DCVs and cargo is coordinately regulated, allowing secretory cell types to adjust their output in response to nutrient (e.g. glucose) and endocrine signals (e.g. glucagon) (reviewed in [1],[2]). The molecular and cellular mechanisms by which neuroendocrine cells achieve this coordinated secretory regulation are poorly understood.

Recent work suggests that the DCV-associated protein Islet Antigen-2 (IA-2, a.k.a ICA512) and its paralog, IA-2β (a.k.a. Phogrin), might hold clues to these regulated secretory processes. IA-2 and IA-2β are members of a family of protein tyrosine phosphatases (PTP) that lack catalytic activity [3]–[8]. Both IA-2 and IA-2β are transmembrane proteins localized to DCVs and are present in most, if not all, neuroendocrine tissues [6],[9]. Mouse knockouts of either IA-2 or IA-2β result in impaired glucose-induced insulin secretion and elevated responses to glucose tolerance tests [10],[11]. The IA-2 and IA-2β double knockout mice have enhanced phenotypes, including glucose intolerance [12] and infertility in female mice due to reduced luteinizing hormone secretion [13]. Over expression of IA-2 in insulin-expressing MIN6 cells markedly increases glucose- or K^+^-induced insulin secretion, the number of secretory vesicles, the levels of vesicle-associated proteins (such as synaptotagmin and VAMP2), and the insulin content suggesting that IA-2 might be involved in the stabilization of DCVs [14]. Finally, recent work suggests that IA-2 can be cleaved in pancreatic β-cells upon glucose stimulation. This cleavage releases a cytoplasmic tail of IA-2 that modulates the activity of STAT5, a transcription factor regulating the transcription of the insulin gene and other DCV components [15]. Together, these results implicate IA-2 and IA-2β as key players in DCV and associated cargo abundance in pancreatic β-cells.

To further explore the role of IA-2/IA-2β in neuroendocrine secretion, we have studied its functions in the model organism *C. elegans*. *C. elegans* is a genetically tractable system with a simple nervous system (302 neurons) that has made major contributions to our understanding of synaptic vesicle (SV) [16],[17] and DCV trafficking and neuroendocrine secretion [18]–[21]. It has previously been shown that the gene *ida-1* encodes the single *C. elegans* factor related to both mammalian IA-2 and IA-2β, and that IDA-1 is an evolutionarily conserved neuroendocrine protein that is involved in acetylcholine release and functions in the insulin-like signaling pathway [7],[18],[22]. Our DCV-specific IDA-1::GFP integrated transgenic strain (KM246) has provided a unique marker for studying DCV function using internal reflection fluorescence microscopy and direct electrophysiological assays [21].

We report here on the characterization of a mutant isolated in a genetic screen for abnormal patterns of IDA-1::GFP reporter gene expression. The identified allele, *gv560*, results in markedly increased levels of gene expression for multiple DCV components, increased DCV numbers, and exhibits several behavioral phenotypes linked to increased neuronal secretion. This mutation maps to the gene *pag-3*/*Gfi-1*, which encodes a zinc-finger transcription factor that functions as a repressor in many biological systems (reviewed in [23]). We further show that the effects of *pag-3* mutations on DCV biosynthesis are IDA-1-dependent, suggesting that PAG-3-mediated repression is part of a regulatory mechanism governing DCV numbers and cargo release.

## Results

### A Mutation in *pag-3* Results in the Up-Regulation of IDA-1::GFP

**Figure 1:**
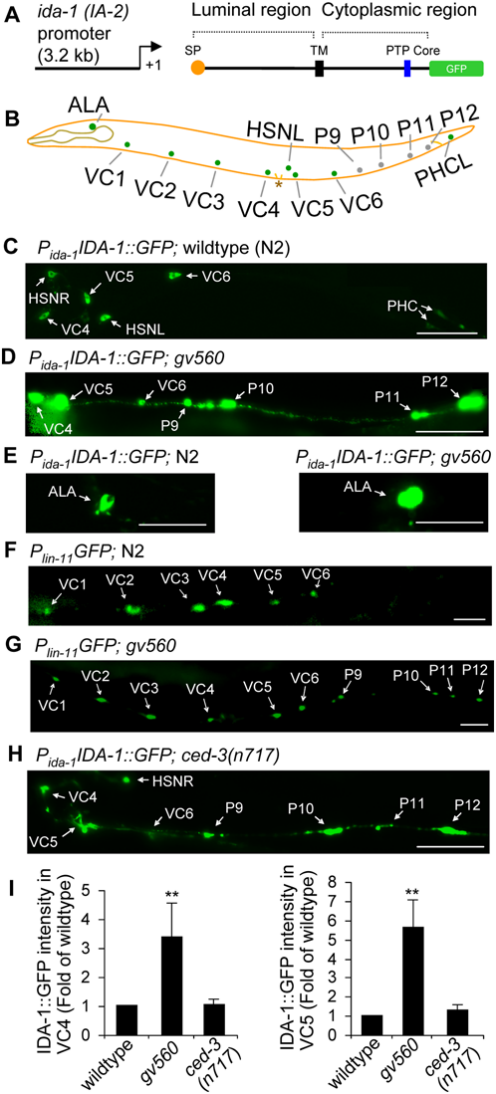
Up-regulation of the IDA-1::GFP translational reporter gene in normal and ectopic neurons in *gv560* mutant animals. (A) Schematic diagram of the integrated IDA-1::GFP translational fusion reporter transgene in strain KM246 that was used for genetic screens. SP, signal peptide; TM, transmembrane domain; PTP, protein-tyrosine phosphatase-like domain. (B) Schematic diagram of a *C. elegans* hermaphrodite showing the positions of relevant cell bodies from the left lateral view with anterior to the left and dorsal to the top. (C) Ventral view of IDA-1::GFP transgene expression in VC4, VC5, VC6, PHC, and HSN neurons in wild type animals. (D) IDA-1::GFP levels in *gv560* mutant animals is markedly increased in VC4 and VC5 neurons and also is observed in four additional neurons (P9–P12 descendants) in the posterior region. (E) IDA-1::GFP in the cell body of the ALA neuron in the head of a wild type (left panel) and *gv560* mutant (right panel) animal; images were collected with identical exposure times. (F) P*_lin-11_*GFP reporter expression in VC1-6 cells in a wild type animal. (G) P*_lin-11_*GFP reporter expression confirmed that the additional posterior neurons observed in *gv560* mutants are derived from P9–P12. (H) IDA-1::GFP expression is observed in extra neurons derived from P9–P12 in *ced-3* mutant animals, but, it is not upregulated in VC or HSN neurons (only HSN(R) is visible in this focal plane). (I) Average intensities of IDA-1::GFP for five VC4 (left) or VC5 (right) neurons in each strain were quantitated and compared between wild type controls and *gv560* or *ced-3(n717)* mutants. **, P<0.01. All images are of adult animals oriented with anterior to the left and (where possible) dorsal towards the top. Scale bars indicate 40 µm.

In order to investigate the molecular mechanisms regulating DCV biosynthesis and turnover *in vivo*, we screened a mutagenized population of *C. elegans* animals for altered patterns of IDA-1::GFP (Figure 1A–E). The wild type IDA-1::GFP reporter strain we used (KM246) harbors a full length translational fusion of IDA-1 to GFP driven by a portion of its own promoter (Figure 1A) that results in strong *gfp* expression in a subset of larval and adult neurons; the endogenous distribution of IDA-1 is more widespread than this particular reporter, and is perhaps ubiquitous within all neurons and neurosecretory cells of the animal [18],[24]. Specifically, we observed GFP in the following neurons in strain KM246 at the frequencies indicated parenthetically: ALA (a single neuron of unknown function that extends a processes the length of the body (100%)), VCs (hermaphrodite specific **V**entral **C**ord motor neurons involved in egg laying (100% VC4&VC5; 31% VC6; 26% for VC1-3), the HSNs (**H**ermaphrodite **S**pecific **N**eurons regulating egg laying (29%)), and PHCs (tail neurons, possibly mechanosensory (18%)) (Figure 1B, C, E) [18]. A screen of 4,400 haploid genomes revealed three mutations that significantly altered this reporter gene expression pattern; two had additional developmental defects and were not maintained. The remaining mutant allele, *gv560*, was homozygous viable and was fully penetrant for multiple phenotypes, including significantly up-regulated levels of IDA-1::GFP levels in a subset of neurons where it is normally observed (Figure 1B–E). Some *gv560* mutant neurons consistently displayed increased levels of GFP (eg. VC4, VC5, and ALA) whereas other neurons (eg. VC1∼3, VC6, HSNs, and PHCs) had a more variable pattern. These differences roughly correlated with the degree of mosaic expression normally observed for this integrated transgene in a wild type background, suggesting these differences might simply reflect the functional efficiency of the promoter element used in this particular reporter. In addition, the *gv560* allele resulted in extra presumptive neurons along the posterior ventral midline (Figure 1D) and an uncoordinated (Unc) phenotype, particularly when moving in the reverse direction. All *gv560* phenotypes were recessive.

The location and appearance of the extra neurons in *gv560* animals suggested they might be surviving P cell lineage descendants that normally are eliminated during larval development by cell death [25]. A reporter gene driven by the *lin-11* promoter (P*_lin-11_*::GFP) has previously been used to identify these neurons in cell death mutants [26]. We crossed *gv560* mutant animals into a strain harboring P*_lin-11_*::GFP. While the P*_lin-11_*::GFP is only present in VC1∼VC6 neurons in wild type animals (Figure 1F), we found that this reporter was also active in many of the extra *gv560* mutant cells (Figure 1G)[26]. This result strongly suggested that most, if not all, extra neurons observed in homozygous *gv560* animals were VC-like neurons derived from Pn.aap-like cell lineages that had failed to undergo normal programmed cell deaths. The failure of programmed cell deaths in *ced-3* mutants also results in extra VC-like neurons [27]. Therefore, we crossed *ced-3(n717)* mutants with our IDA-1 reporter and assayed GFP levels. IDA-1::GFP was not increased in the VC or VC-like neurons of *ced-3* mutants when compared to wild type animals (Figure 1H). Quantitative analysis of IDA-1::GFP intensity in VC4 and VC5 neurons showed that the GFP signal in *gv560* is ∼3.4-fold and ∼5.6-fold of wild type respectively, while the GFP signal in *ced-3* mutant is similar to wild type (Figure 1I).

**Figure 2:**
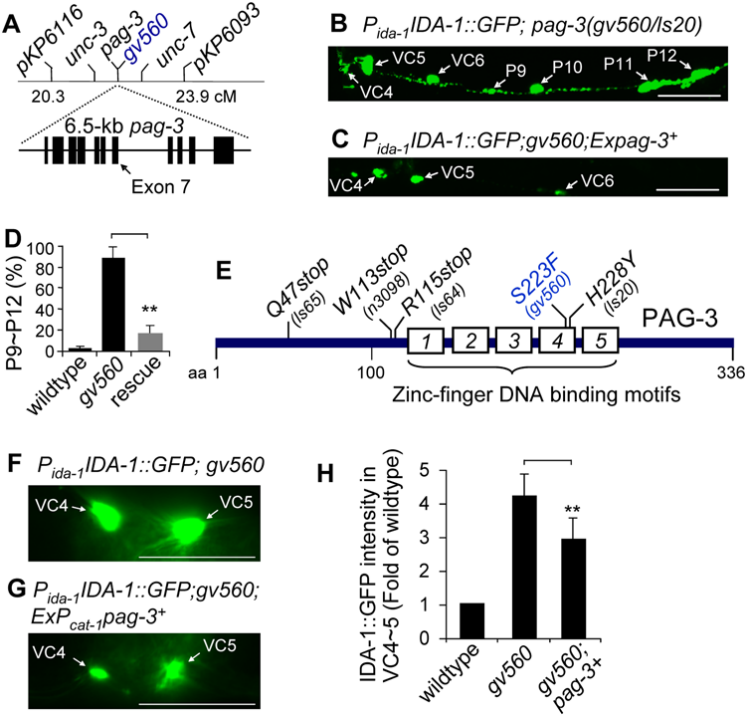
The *gv560* mutation is an allele of the gene *pag-3*, which encodes a zinc-finger transcription factor. (A) The genetic interval in which *gv560* was mapped genetically is shown with a 6.5-kb stretch of genomic DNA from the region shown below. The 11 exons of *pag-3* (boxes) were sequenced to identify the *gv560* allele as a C to T mutation resulting in a change in an evolutionarily conserved amino acid (S223F) in exon 7 as indicated by an arrow. (B) Trans heterozygous *gv560/pag-3(ls20)* animals showed the same phenotypes (IDA-1::GFP up regulation and extra neurons) as either the *gv560* or *pag-3(ls20)* homozygous single mutants (see Figure 1D). (C) *pag-3(gv560)* phenotypes are rescued by a 6.5-kb PCR product that contains the putative promoter and all exons and introns of the wild type *pag-3* gene. In both panels B and C, the mid-section of an adult animal is shown with anterior to the left and dorsal at top, each imaged with identical exposure times. Scale bar represents 40 µm. (D) The percent of P9–P12 derived cells expressing the IDA-1::GFP transgene was compared in N2, *gv560*, and the rescued strain by counting 100 worms in each group, demonstrating nearly full rescue by the wild type *pag-3* transgene (**, P<0.01). (E) The *gv560* allele results in a S223F mutation in the fourth zinc-finger region. Several previously identified alleles of *pag-3* are also indicated. (F–H) Cell autonomous rescue of the GFP up regulation phenotype with a wild type encoding PAG-3 transgene. Representative ventral view images of P*_ida-1_*IDA-1::GFP levels in VC4 and VC5 (anterior to the left) in *pag-3(gv560)* animals (F) and animals harboring a potentially rescuing transgene (G) using identical exposure conditions. Partial rescue was achieved by expressing the full-length *pag-3* cDNA under the *cat-1* promoter (−3 kb) from an extrachromosomal transgene. The P*_cat-1_* and P*_ida-1_* promoters used in these experiments have overlapping activity in VC4, VC5, and the HSNs, the only sites for which partial rescue was observed, demonstrating cell autonomy for PAG-3 activity. (H) Quantitative analysis of GFP levels in VC4 and VC5 neuron cell bodies from images of 15 animals for each strain using NIH ImageJ software. On average, animals harboring an extrachromosomal rescuing *pag-3* transgene had a ∼31% (P<0.01) reduction in the levels of IDA-1::GFP in VC4 and VC5 when compared to non-rescued mutants; ALA neuron GFP intensity was not altered in the rescued animals (not shown). Scale bar represents 40 µm.

Using genetic and single nucleotide polymorphism (SNP) markers, we mapped the *gv560* mutant to the right end of the X chromosome (Figure 2A) (see Materials and Methods). There are three known mutations in this region that result in an Unc phenotype; *unc-3*, *pag-3*, and *unc-7*. One of these, *pag-3*, has previously been shown to result in extra VC-like neurons due to cell lineage alterations [26]. We found that *gv560* failed to complement two different *pag-3* alleles (Figure 2B). Moreover, a 6.5 kb genomic fragment from the *pag-3* genomic locus rescued the *gv560* mutant phenotypes (Figure 2C, D), indicating that *gv560* is a mutant allele of *pag-3*.

The *pag-3* gene encodes a zinc-finger (Zn-finger) transcription factor that was originally identified in *C. elegans* due to altered gene expression patterns in specific neurons (**pa**ttern of **g**ene expression abnormal, *pag-3*) [28]. More recently, *pag-3* mutations have been shown to affect cell fate decisions within certain lineages resulting in the generation of additional posterior neurons [26]. Sequence analysis of the *pag-3* gene in the *C. elegans gv560* mutant animals revealed a C to T transition in exon 7 resulting in a S223F amino acid change within the fourth Zn-finger coding region (Figure 2E). Multiple sequences alignment showed that S227 in PAG-3 is evolutionarily conserved from *C. elegans* to humans (data not shown). Examination of two previously identified *pag-3* alleles (*is20* and *n3098*) (Figure 2E), demonstrated that each had the same phenotype as *gv560* with respect to IDA-1::GFP levels, either alone or in trans-heterozygous combination with *gv650* (Figure 2B). Because *pag-3(ls20)* (Figure 2E) has previously been shown to behave genetically as a null allele [28], we conclude that *gv560* is also genetically null; we can not eliminate the possibility that it retains some molecular function.

To determine if PAG-3 activity acted cell autonomously, we sought to rescue the GFP over expression phenotype in a subset of neurons expressing IDA-1::GFP in the *pag-3(gv560)* mutants. The *cat-1* gene encodes a synaptic vesicular monoamine transporter [29] that has been reported to be expressed in several neurons (Shawn Lockery, personal communication; http://chinook.uoregon.edu/promoters.html), including VC4, VC5 and the HSNs where we see IDA-1::GFP phenotypes in the *pag-3(gv560)* mutant. We confirmed this expression pattern using a ∼3 kb region upstream of the *cat-1* coding region to drive GFP (data not shown); this reporter was not expressed in ALA. The P*_cat-1_* promoter was placed upstream of a wild type, full-length cDNA encoding PAG-3 and used to make stable, extrachromosomal transgenic strains in the P*_ida-1_*::IDA-1::GFP; *pag-3(gv560)* mutant background. Interestingly, we found that this transgene was often toxic, resulting in several strains in which most transgenic animals arrested at the L1 stage of development and subsequently died. However, we were also able to generate multiple, independent stable strains in which transgenic animals grew normally, presumably reflecting expression of the transgene at a sub-toxic dose. We scored GFP levels in these healthy strains, focusing on the VC4/5 versus ALA over expression phenotypes because these cells always express IDA-1::GFP. A visual screen revealed that 28% (n = 100) of P*_ida-1_*IDA-1::GFP; *pag-3(gv560)* mutants harboring the P*_cat-1_*::PAG-3 transgene had a reduction in VC4/5 GFP to near wild type levels whereas GFP over expression in ALA remained unaffected (Figure 2F, G). We did not expect full rescue as the extrachromosomal rescuing transgene is mitotically unstable. A quantification of GFP intensity in VC4 and VC5 for 15 selected animals of each strain similarly showed a reduction (31%) in average intensity in the rescued strain (Figure 2H). These results demonstrated that wild type PAG-3 activity in VC4 and VC5 was likely acting cell autonomously, with no change in the GFP over expression phenotypes seen in neurons that did not express the P*_cat-1_*::PAG-3 transgene.

**Figure 3:**
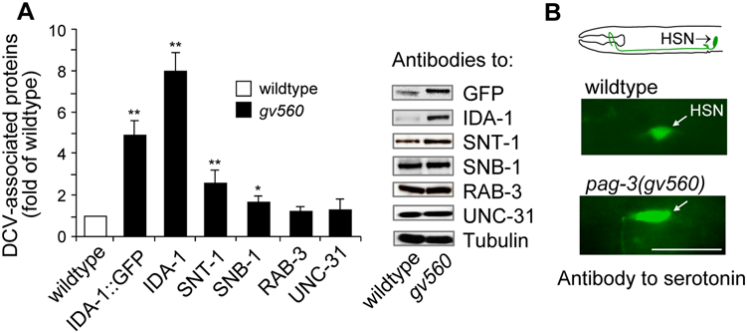
Changes in IDA-1 and other dense core vesicle proteins in *pag-3(gv650)* mutants. (A) Transgenic IDA-1::GFP and endogenous IDA-1 levels in *gv560* mutants are elevated 5-fold and 8-fold, respectively, in *pag-3(gv650)* mutants compared to wild type animals as determined by Western blot analysis. Quantitative analysis is shown to the left and blot signals to the right. SNT-1 and SNB-1 in the *gv560* mutant are increased 2.9-fold and 1.8-fold, respectively, whereas RAB3 and UNC-31 levels remained unchanged. Tubulin levels served as the protein loading control. **, P<0.01, *, P<0.05. (B) The DCV cargo component serotonin is elevated 2.5-fold (P<0.01) in the HSN neurons of *gv560* mutants versus controls as shown by immunofluorescent staining of whole animals with an anti-serotonin antibody; exposure times and settings for both images was identical. As indicated in the diagram at top, the adult animals imaged are oriented with anterior to the left and dorsal to the top. Scale bar represents 20 µm.

To quantify the increased IDA-1::GFP levels in *pag-3(gv560)* mutants, equal amounts of protein extract from *gv560* animals were compared to the parental reporter strain on Western blots probed with anti-GFP and anti-IDA-1 antibodies. We found that GFP levels in the *gv560* mutant were approximately five times higher than that in the parental controls when internally compared to a tubulin protein control (Figure 3A). Interestingly, the IDA-1 peptide containing protein levels in *gv560* mutant, derived from both the endogenous gene and the reporter fusion transgene, were nearly eight-fold higher than in controls. This demonstrated that the changes in the reporter accurately reflected alterations in endogenous IDA-1 protein levels and not merely changes in the reporter gene expression.

### *pag-3* Mutations Result in the Up-Regulation of Multiple DCV Components

To probe whether the effect of *pag-3(gv560)* was limited to IDA-1 protein or more generally applicable, we assayed five additional neurosecretory components for which specific *C. elegans* antibodies were available: (1) synaptotagmin (SNT-1), an integral membrane protein of synaptic vesicles (SVs) [30] that has also been implicated in DCV cargo release [31]; (2) synaptobrevin (SNB-1), an integral membrane protein of SVs implicated in neurotransmitter release [32] and for which mammalian data demonstrates its presence in DCVs [33]. (3) RAB-3, a member of the Ras GTPase superfamily that regulates the axonal distribution of SVs and DCVs and, consequently, neurosecretion [22],[34]; (4) UNC-31, a **c**alcium-**a**ctivated **p**rotein for **s**ecretion (CAPS) homolog required for DCV docking and cargo release (Livingstone, 1991, Ph.D. Thesis) [21],[35],[36]; and (5) serotonin, a DCV cargo component present in HSN neurons and several neurons in the head region [37]. We found that the levels of SNT-1 and serotonin are substantially increased in *gv560* mutants (Figure 3A, B), whereas the SNB-1 level is more moderately increased. In contrast, the level of proteins lacking vesicle transmembrane motifs (e.g. RAB-3 and UNC-31) and the control (α-tubulin) did not change in the *gv560* mutant (Figure 3A).

It was possible that the increases in endogenous IDA-1, SNT-1, and serotonin observed in *pag-3(gv560)* mutants were due to the extra VC-like (and perhaps other undetected) neurons in these mutants. To address this, we checked protein levels in the mutant *ced-3* that also results in extra VC-like neurons (and other cell types) due to a lack of programmed cell death [27]. There was no detectable increase in any assayed protein in *ced-3(n717)* mutants compared to wild type (data not shown), suggesting that observed protein increases in the *pag-3(gv560)* mutant are due to up-regulation or increased stability of these factors rather than the increased neuronal cell number.

**Figure 4:**
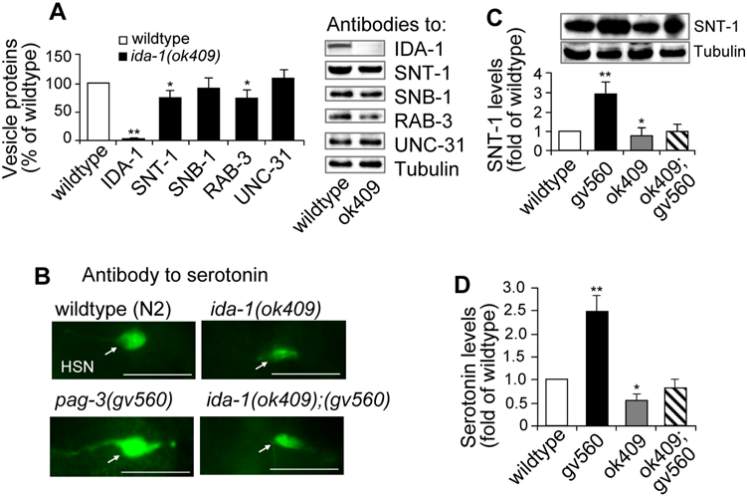
Increased levels of vesicle proteins in the *pag-3(gv650)* mutant are dependent on wild type IDA-1 activity. (A) Complete loss of IDA-1 activity due to the *ida-1* null deletion allele *ok409* results in a moderate, but significant, reduction in the levels of some vesicle-associated proteins (SNT-1 and RAB-3, *P<0.05) as indicated quantitatively at left and by Western blot signal to the right. All values are given as a percentage of wild type that is arbitrarily set to 100% as indicated by an open box. Error bars indicated variation of individual antibody level. (B) The level of the vesicle cargo component serotonin was compared in HSN neurons by immunofluorescent staining in the indicated four strains. Images show close up of HSN cell body of an adult animal orientated with anterior to the left and dorsal to the top. Exposure times were identical for all images. Scale bar represents 20 µm for all images. (C) The SNT-1 levels were compared by Western blots (shown at top and quantified below) in four different mutant backgrounds as indicated. Note that the increased SNT-1 in *gv560* mutants (**, P<0.01) is reduced to wild type levels when the IDA-1 activity is removed in the *ida-1(ok409);pag-3(gv560)* double mutants. (D) Quantitative analysis of the images show in panel C confirmed that the signal intensity increases seen in the *gv560* mutant background are reduced to wild type levels in the *ida-1(ok409);pag-3(gv560)* double mutants (**, P<0.01). Note that the double mutant levels are not identical to *ida-1(ok409)*, suggesting there may be a small, IDA-1-independent effect of the *pag-3* mutation on serotonin.

The significantly increased IDA-1 protein levels in the *pag-3(gv560)* mutant prompted us to explore DCV components more thoroughly in *ida-1* mutants alone. We assayed by Western blot the levels of DCV-related proteins (IDA-1, SNT-1, SNB-1, RAB-3, and UNC-31) in wild type animals and an *ida-1(ok409)* mutant using tubulin as a control (Figure 4A). IDA-1 was undetectable, as expected, in the predicted null allele of *ida-1* [18]. Of the remaining proteins tested, SNT-1 and RAB3 showed a slight reduction (∼80% of the N2 control, p<0.05) in *ida-1(ok409)* mutants (Figure 4A). We also assayed serotonin levels in *ida-1(ok409)* mutants as an example of a DCV cargo component and found a significant reduction (∼50% of the N2 controls, p<0.05) (Figure 4B). Interestingly, the increased levels of SNT-1 and serotonin found in *pag-3(gv560)* animals are also eliminated when IDA-1 activity is removed (Figure 4B–D). A comparison between *ida-1(ok409);pag-3(gv560)* double mutant protein levels to *ida-1(ok409)* mutants alone demonstrates that *ida-1* is epistatic to *pag-3* for the change in both SNT-1 and serotonin. We concluded that loss of PAG-3 activity results in an up-regulation of at least some vesicle-related components and that this up-regulation also requires functional IDA-1.

### *pag-3(gv560)* Mutants Have Altered Gene Expression that is not *ida-1*-Dependent

Previously described roles of PAG-3/Gfi-1 and related proteins in transcriptional repression (see review in [23]), coupled with the increased levels of DCV component proteins in the *gv560* mutant, led us to examine DCV and SV component gene expression in these mutant animals. We selected four genes whose products are DCV-associated proteins, four genes that encode DCV cargo proteins, four genes expected to function primarily in SV pathways, and three non-vesicle related genes as controls (Table 1). We used quantitative real-time polymerase chain reaction (RT-PCR) assays to measure the expression of these 15 genes in young adult wild type and *pag-3(gv560)* mutant animals relative to internal controls. We found that *pag-3(gv560)* mutants showed significantly increased expression of three genes (Table 1), including two that encode DCV-membrane associated proteins (IDA-1 and SNT-1) and one encoding DCV cargo related protein (INS-1 or insulin-like molecule 1). There were no changes in the expression of SV genes and the three non-vesicle associated genes. Interestingly, the DCV membrane-associated gene *ida-1* showed the greatest magnitude of change in *pag-3(gv560)* mutants (Table 1).

**Table 1:** mRNA Levels of Vesicle-related Genes Assayed by RT-PCR.

*C. elegans*	Mammalian	Relative Ratio (*gv560*/N2)	References
**Encoding DCV membrane-associated proteins**			
*arf-6*	ARF6	1.02±0.01	[77]
*ida-1*	IA-2	8.55±0.03	[7]
*rab-3*	RAB3	0.97±0.04	[34]
*snt-1*	Synaptotagmin	4.13±0.63	[30]
***Encoding DCV cargo***			
*egl-3*	PC2	1.19±0.03	[78]
*flp-1*	FMRFamide 1	0.98±0.05	[79]
*ins-1*	Insulin-like gene 1	3.04±0.19	[80]
*nlp-1*	Neuropeptide-like	0.92±0.06	[81]
***Encoding SV membrane-associated proteins***			
*snb-1*	Synaptobrevin I	1.01±0.08	[32]
*snn-1*	Synapsin	0.90±0.11	[82]
*sph-1*	Synaptophysin	1.03±0.06	[83]
*unc-26*	Synaptojanin	1.11±0.19	[83]
***Encoding non-vesicle proteins***			
*act-1*	Actin	1.15±0.02	[84]
*myo-1*	Myosin	1.08±0.03	[84]
*tba-2*	Tubulin, α1	1.06±0.05	[85]

Total RNA was prepared from young adult N2 or *pag-3(gv560)* animals.

Because the up-regulation of at least some of the vesicle-related proteins in *pag-3(gv560)* is dependent on the presence of wild type IDA-1, we thought it possible that a feedback mechanism could link vesicle biogenesis, utilization, or stability to gene expression. Previous work has shown that a cleaved cytosolic tail of mammalian IA-2/IDA-1 interacts and modulates the transcription factor STAT-5 that, in turn, positively regulates insulin gene expression [15]. This link between IA-2 and the transcription of a DCV cargo-encoding gene in mammals prompted us to investigate in *C. elegans* the role of *ida-1* and *sta-1* (encoding the lone STAT-related factor) in DCV homeostasis. We first examined the mRNA levels of all 15 genes listed in Table 1 by quantitative real-time PCR in *ida-1* mutants; we found no significant differences compared to wild type levels. We also crossed *ida-1(ok409)* into the *pag-3(gv560)* mutant background and repeated the gene expression analysis. We found that the mRNA levels in the *pag-3(gv560);ida-1(ok409)* double mutants remained the same as in the *pag-3(gv560)* single mutant (except for *ida-1* itself, data not shown), demonstrating that transcriptional alterations caused by loss of PAG-3 are not dependent on IDA-1 activity. Finally, we crossed the presumptive null deletion allele *sta-1(ok587)* into the IDA-1::GFP reporter strain, either alone or in combination with *pag-3(gv560)*, and assayed IDA-1 and IDA-1::GFP protein levels. The absence of STA-1 activity did not change any IDA-1 protein phenotypes. Our data strongly suggest that the presence or absence of IDA-1 alone has no effects on the transcriptional regulation of the DCV and SV genes we examined in *C. elegans* nor does the absence of STAT activity influence vesicle-associated protein levels. Because the up-regulation of DCV components observed in *pag-3(gv560)* is dependent on wild type IDA-1 activity, whereas changes in gene expression are not, we conclude that the role of IDA-1 in DCV regulation is post-transcriptional.

### The Molecular Changes of *pag-3(gv560)* Correlate with Altered Neurosecretion

**Figure 5:**
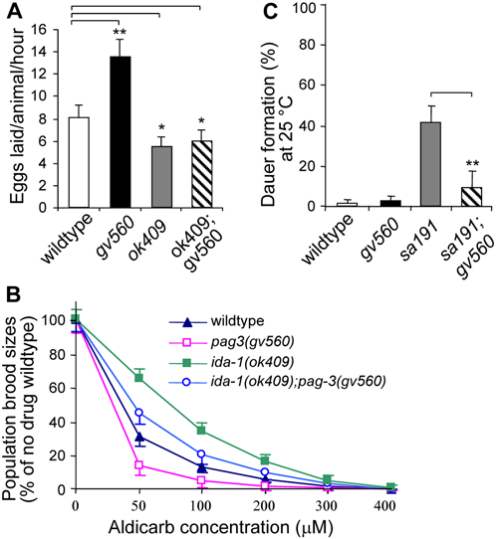
Neurosecretory phenotypes of *pag-3(gv650)* are enhanced and are dependent on wild type IDA-1 activity. (A) An egg-laying assay was used to monitor neurosecretory behavior in wild type and mutant animals as described in the text. The average number eggs laid in *pag-3(gv560)* mutant animals is much higher than that in wild type animals (**, p<0.01), indicative of increased neurosecretion in the *pag-3* mutant. Both *ida-1(ok409)* single mutant and *ida-1(ok409);pag-3(gv560)* double mutants laid less eggs than wild type controls (*, p<0.05), demonstrating that *ida-1* is epistatic to *pag-3* for this phenotype. Data presented as the mean±SE (n = 10). (B) *pag-3(gv560)* mutants are hypersensitive to aldicarb. Shown are the aldicarb dose-response curves for wild type (N2), *pag-3(gv560)*, and *ida-1(ok409)* single mutants and *ida-1(ok409);pag-3(gv560)* double mutants. Increased neurosecretion makes animals hypersensitive to aldicarb (reduced survival), as is seen in the *pag-3(gv560)* mutant. The hypersensitivity of *pag-3(gv560)* animals is suppressed to below wild type levels in the *ida-1(ok409);pag-3(gv560)* double mutants. The one hundred percent value represents the number of progeny produced from a starting population of wild type L1 larvae over a 96 hr period in the absence of aldicarb. Curves are representative of duplicate experiments in three independent assays. (C) Wild type animals or *pag-3(gv560)* mutants alone have no dauer phenotype at 25°C compared to the *daf-28(sa191)* temperature sensitive allele that gives rise to ∼43% dauers at this temperature. The *pag-3(gv560)* mutation suppresses the *daf-28(sa191)* mutant dauer phenotype from ∼43% to ∼9% (p<0.01).

To determine if the changes in gene expression and protein levels observed in *pag-3(gv560)* mutants had behavioral consequences, we tested two behaviors that reflect vesicle (DCV and SV) cargo release: egg-laying and aldicarb resistance. Egg (embryo) laying by gravid hermaphrodites is a complex neuroendocrine behavior involving serotonin and acetylcholine released by VC and HSN neurons acting on the vulval muscles [38]. The rate of egg laying is dependent on many variables, including animal age, population density, and food supply. To eliminate some of these variables, individual young gravid hermaphrodites were picked into single wells of a 96-well micro titer plate containing M9 buffer. Exogenous serotonin (12.9 mM) was added to stimulate wild type egg laying to a rate (∼8 eggs/hr/animal) that could be quantified easily within a 60 minute assay and various mutant animals were tested. We found that *pag-3(gv560)* mutants had a significant increase (∼34%) in egg-laying compared to wild type controls (Figure 5A). In comparison, *ida-1(ok409)* single mutants and *ida-1(ok409);pag-3(gv560)* double mutants had a ∼33% and ∼26% reduction, respectively, in egg laying compared to wild type controls. As observed for protein level changes, the egg-laying phenotype of *gv560* was dependent on wild type IDA-1 activity. To control for possible effects of the extra VC-like neurons present in *gv560* animals on egg-laying, we also tested laying rates in *ced-3(n717)* mutants that also have extra VC-like neurons; *ced-3* mutant rates of egg laying were comparable to wild type animals (data not shown). These results suggested that increased neurosecretion from the HSNs, and/or the VCs, and not the presence of extra VC-like neurons, likely underlies the enhanced egg-laying phenotype of *gv560* mutants in response to serotonin.

As a second test of neurosecretion, we used the acetylcholinesterase inhibitor aldicarb to assess acetylcholine (Ach) release and presynaptic function [39],[40]. Ach release is traditionally considered an assay of SV function, not DCVs. However, it has been previously shown that disruption of DCV function impaired, directly or indirectly, the release of Ach [18],[35],[41],[42]. We used a population growth rate assay in which we counted the number of progeny from parental animals after 96 hr of growth on plates containing various concentrations of aldicarb [18],[39]. This assay provides a more sensitive and quantitative measure of aldicarb sensitivity compared to scoring paralysis alone. We found that *pag-3(gv560)* mutants showed greater sensitivity to aldicarb than wild type animals (Figure 5B), suggesting that these mutants release more Ach than wild type controls. In contrast, the *ida-1(ok409)* mutant animals showed resistance to aldicarb as previously reported [18] due to disruption of DCV function and decreased neurosecretion. Again, the *gv560* aldicarb phenotype was suppressed in double mutant combination with *ida-1(ok409)*. The increased rates of egg-laying and increased sensitivity to aldicarb observed for *pag-3(gv560)* are both consistent with enhanced neurosecretion in these mutant animals.

We also investigated the role of *pag-3* in dauer formation, an alternative part of the life cycle in *C. elegans* that is regulated (in part) by an insulin-like signaling pathway dependent on proper neuroendocrine secretion. We compared dauer formation in wild type, *pag-3(gv650)* and temperature-sensitive (ts) *daf-28(sa191)* mutants, each reared at an elevated temperature of 25°C. The wild type and *pag-3* mutant animals showed no appreciable dauer formation at this temperature (Figure 5C). In contrast, the *daf-28* mutant harboring a ts allele of the insulin-like protein it encodes resulted in 40% dauers due to reduced signaling through the insulin-like signaling pathway at this temperature [43]. We found that the *daf-28(sa191);pag-3(gv650)* double mutant resulted in a ∼75% reduction in dauer formation at 25°C compared to *daf-28(sa191)* alone (Figure 5C) demonstrating enhanced signaling through the pathway. This result is consistent with our egg-laying and aldicarb assays and together suggested that loss of PAG-3 activity results in enhanced neuroendocrine secretion affecting many cell types.

### Presynaptic DCV Numbers are Up-Regulated in *pag-3(gv560)* and Down-Regulated in *ida-1(ok409)* Mutants

**Figure 6:**
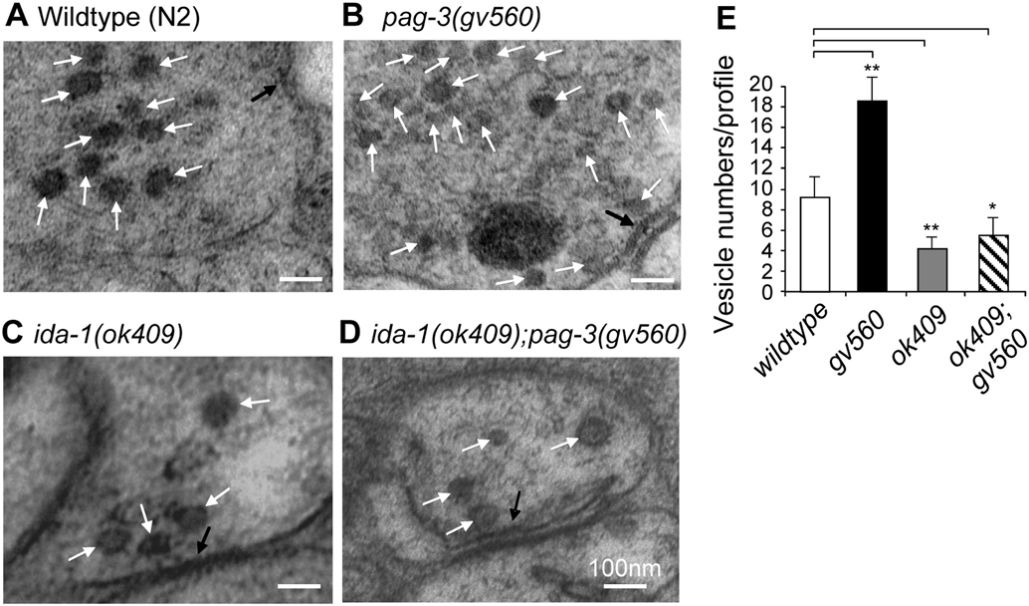
Changes in the presynaptic region number of dense core vesicle in *pag-3* and *ida-1* mutants. Electron micrographs of unidentified ventral cord neurons showing the characteristic presynaptic density (black arrows) from wild type animals (A), *pag-3(gv560)* mutants (B), *ida-1(ok409)* mutants (C), and *ida-1(ok409);pag-3(gv560)* double mutants (D). Large dense core vesicle-like structures are indicated by white arrows in all panels. The scale bar represents 100 nm for all images. (E) The average number of DCVs per presynaptic region was quantified relative to wild type controls. The *pag-3(gv560)* mutants have an increased number of DCVs, whereas the *ida-1(ok409)* single mutants and *ida-1(ok409);pag-3(gv560)* double mutants have significantly decreased numbers (**p<0.01, *p<0.05). Note also that the DCVs in *pag-3(gv560)* mutants (panel B) are consistently smaller in size and often irregularly shaped. Error bars are one standard deviation.

Previous cell culture work showed that DCV numbers are increased 2∼3-fold following over expression of mammalian IA-2 in mouse MIN6 cells, as assayed by vesicle counts from electron micrographs [14]. We have demonstrated here that IDA-1/IA-2 is up-regulated in *pag-3* mutants, perhaps similarly resulting in increased DCV numbers and increased neurosecretion. Although technically challenging in *C. elegans*, we sought to determine by electron microscopy if the overexpression of *ida-1* observed in *pag-3(gv560)* mutants was accompanied by increased numbers of DCVs per neuron. Wild type and mutant animals were fixed, sectioned and examined by electron microscopy (see Materials and Methods). We focused on the ventral nerve cord region and identified presynaptic regions by serial sections; individual neurons in any section were not precisely identified. We found that the average density of presynaptic region DCVs in *pag-3(gv560)* was about twice the value of that in wild type controls (Figure 6A, B, E). We also quantitated DCV numbers in *ida-1(ok409)* mutants and found a 50% reduction compared to wild type (Figure 6C, E). Of particular interest, we found that the *pag-3(gv560);ida-1(ok409)* double mutants eliminated the increased DCV numbers observed in the *pag-3(gv560)* single mutant (Figure 6D, E). Our results demonstrate that presynaptic DCV numbers correlate with neurosecretion-based phenotypes in the mutants studied. That is, *pag-3(gv560)* mutants have increased numbers of presynaptic DCVs in ventral cord neurons and display phenotypes of enhanced egg-laying, enhanced aldicarb sensitivity, and enhanced signaling through the insulin-like pathway. All of these enhanced phenotypes are dependent on wild type IDA-1 activity.

## Discussion

We have capitalized on the genetics of *C. elegans* to identify factors that contribute to the regulation of dense core vesicles (DCVs) and neuroendocrine secretion. Using a reporter gene to mark DCVs in live animals, we carried out a genetic screen and identified a mutation that results in both over and ectopic expression of the reporter. The mutation, that behaves genetically as a recessive null allele, results in an amino acid change (S223F) in the fourth zinc finger of the transcription factor PAG-3/Gfi-1. This mutation (*gv560*) results in increased gene expression and levels of several DCV-associated proteins and increased numbers of DCVs in presynaptic regions of ventral cord neurons. These increases correlate with enhanced neurosecretory phenotypes in *pag-3* mutants and are dependent on wild type IDA-1, the lone *C. elegans* PTP-like factor related to mammalian DCV membrane proteins IA-2 and IA-2β. Although mutations in many other genes, such as *unc-4* and *unc-37* [44],[45], *unc-86* [46],[47] and the EGL-46 mammalian homolog INSM1/IA-1 [48]–[50], have been shown to decrease SV and DCV components and numbers in various neurosecretory systems, this is the first report we are aware of in which a mutation results in DCV component up-regulation. The results of this and previous work suggest that DCV homeostasis is regulated by both stimulatory and inhibitory mechanisms that correlate with levels of IDA-1-related proteins.

Our genetic studies demonstrate that the up-regulation of DCV protein components in *pag-3* mutants is dependent on functional IDA-1 activity. It is possible that this dependence is indirect, with IDA-1 and PAG-3 acting in separate pathways of DCV regulation. For example, the enhanced neurosecretory phenotypes of *pag-3(gv560)* could be suppressed by loss of IDA-1 activity due simply the averaged effects (one positive and one negative) of the two individual mutant phenotypes, each acting in different regulatory cascades. We do not favor this explanation for two reasons. First, both *ida-1* transcription and IDA-1 levels are elevated in *pag-3* mutants. Second, where quantifiable, the genetic interactions between *ida-1* and *pag-3* mutants we observe strongly suggest an epistatic relationship. That is, the *ida-1(ok409);pag-3(gv560)* double mutants more closely resemble *ida-1(ok409)* alone (with the exception of gene expression) rather than a quantitative average of *ida-1(ok409)* and *pag-3(gv560)* effects together. These results strongly suggest that both IDA-1 and PAG-3 act in a common pathway to regulate DCV homeostasis.

**Figure 7:**
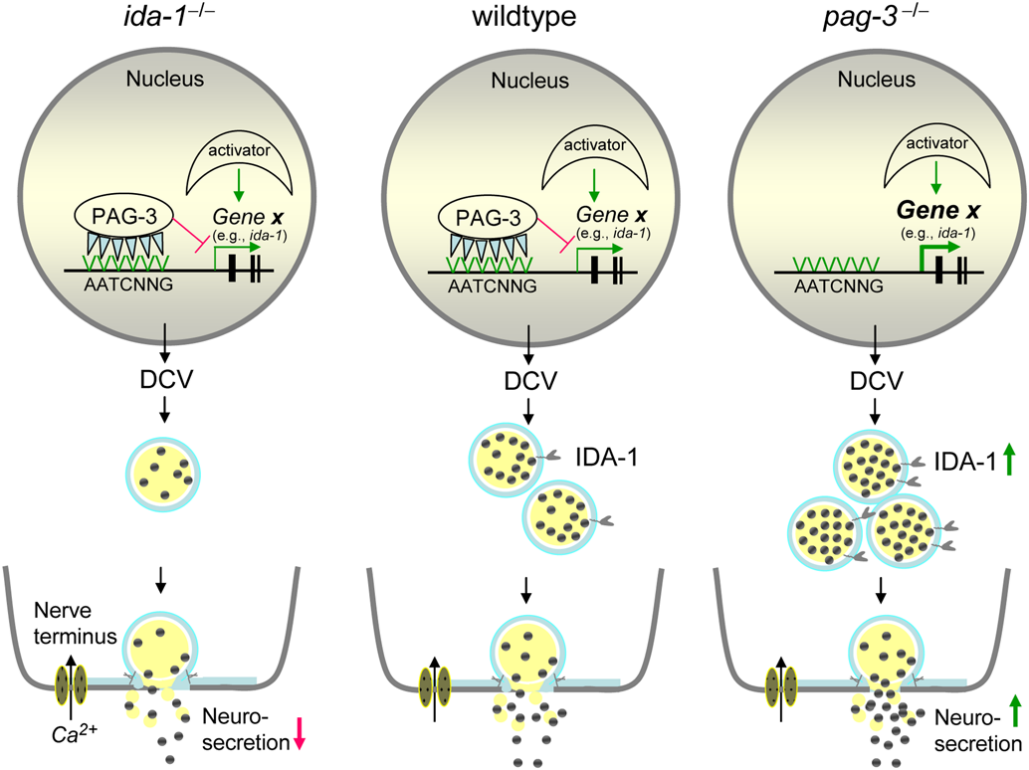
Model of DCV stability by IDA-1. The center schematic shows the wild type scenario in which DCV numbers, cargo, and neurosecretion are regulated by both transcriptional and post-transcriptional mechanisms. *Gene X* is one, or more, hypothetical components or regulators of DCV biogenesis, utilization, or stability. The expression of *Gene X* is controlled by the balanced activities of both positive regulators (activator) and negative regulators, represented here by PAG-3 binding to a canonical Gfi1 promoter sequence (AATCNNG). IDA-1, one candidate for being encoded by *Gene X*, serves to regulate DCV numbers or turnover post-transcriptionally, thus affecting neurosecretory levels. The left panel shows the consequences of loss of IDA-1 in null mutants; DCV biogenesis and/or turnover are altered, resulting in deceased presynaptic DCV numbers and decreased neurosecretion. The right panel shows the effects of loss of the putative negative transcriptional regulator PAG-3. *Gene X* transcription increases with resulting increases in IDA-1, DCV numbers, cargo and neurosecretion, an effect that can be mimicked by increasing IA-2 levels in mammalian cell culture [14].

A model consistent with our results places IDA-1 in an important position in the modulation of DCV numbers and secretion by directly influencing their biogenesis, stability and/or utilization (Figure 7). Actions that increase IDA-1 (e.g. loss of PAG-3) result in increased presynaptic DCV numbers and increased neurosecretion whereas actions that decrease IDA-1 (e.g. loss of IDA-1 itself) result in decreased DCVs and secretion. Loss of both PAG-3 and IDA-1 in double mutant animals results in a DCV phenotype resembling loss of IDA-1. This demonstrates that effects of increased transcription of some DCV component genes, due to loss of PAG-3, are masked by the loss of the post-transcriptional role of IDA-1 in influencing the biogenesis, stability, or utilization of DCVs. The molecular mechanism whereby IDA-1 influences DCV steady state levels is currently unknown and represents an important area for future research. Although IDA-1 activity is an important barometer of DCV homeostasis, it is worth noting that it is not essential for DCV formation and organism viability. Instead, its loss or hyper production in *C. elegans* results in an approximately 2-fold swing, down or up respectively, in DCV numbers. Thus, IDA-1 is part of a larger and as yet undefined, multi-component regulatory system that ensures DCV numbers and cargo reflect the neurosecretory demands of the cell.

Our results are consistent with recent results from vertebrate studies, providing an organismal context for understanding IDA-1/IA-2 function in DCV regulation. Over expression of mouse IA-2 in insulin-expressing MIN6 cells markedly increased induced insulin secretion and the number of DCVs [14]. Studies by Solimena and colleagues have provided evidence that after fusion of the DCV with the plasma membrane during insulin secretion, the cytosolic tail of IA-2 (containing the catalytically inactive PTP-like domain) is cleaved by a calcium-induced and calpain-dependent mechanism [51],[52]. They have proposed that this cleavage event initiates a retrograde signal in which the IA-2 cytosolic domain enters the nucleus to complex with the transcription factors STAT3 and STAT5, thereby blocking their inactivation by de-phosphorylation [15],[52]. In this model, IA-2 is a critical element that links DCV exocytosis to feedback regulation of transcription such that genes encoding IA-2 and other DCV components and cargo are up-regulated upon induced insulin secretion. We find in *C. elegans* that all up-regulated changes in DCV numbers and secretory behaviors due to *pag-3* mutations are ameliorated by loss of IDA-1/IA-2. However, loss of IDA-1 has no effect on the steady state mRNA levels of any vesicle-related genes we assayed (listed in Table 1), nor do we ever detect our IDA-1::GFP translation fusion protein in the nucleus. Thus, functional IDA-1 appears to be necessary post-transcriptionally for the coordinated regulation of DCV biogenesis and utilization, although we have no evidence that reveals the molecular mechanisms by which this occurs. We were unable to link these effects genetically to the single *C. elegans* STAT encoding gene *sta-1*, suggesting that any feedback mechanisms that function in *C. elegans* are not entirely dependent on STAT activity. Similarly, both of the pancreatic β-cell-specific STAT3 and STAT5 knockout mice showed normal insulin content and islet mass [53],[54], suggesting that STAT3 and STAT5 are functionally redundant or are not critical elements for insulin-containing DCV regulation. It is also possible that other transcription factors act redundantly with the STATs, thereby masking the effects of STAT loss of function in this process in both *C. elegans* and mice. Regardless of the role of STAT activity in DCV regulation, our results reveal an additional, post-transcriptional mechanism whereby IDA-1 influences DCV homeostasis.

Our work reveals a novel transcriptional link between IDA-1/IA-2 and DCV regulation that is mediated by PAG-3. PAG-3 is a five C_2_H_2_-type zinc finger transcription factor and it is orthologous to vertebrate Gfi-1 (**g**rowth **f**actor **i**ndependence-1) and *Drosophila* Senseless [28],[55],[56]. In the *C. elegans* nervous system, PAG-3 appears to function primarily as a transcriptional repressor [28], a theme common to the functions described to date for its vertebrate and *Drosophila* orthologs [23],[57]. Interestingly, we find a similar mis-regulation of our IDA-1::GFP reporter gene in *unc-3* mutant animals (unpublished). UNC-3 was recently identified as PAG-3 interacting protein and is an ortholog of the Olf-1/Early B cell factor family of transcription factors involved in neuronal cell development [58]. It is possible that PAG-3 and UNC-3 act together as part of a common transcriptional complex that can influence, directly or indirectly, the expression of genes whose products are necessary for DCV homeostasis. However, the relationship between the activities of these two factors appears to be complex. UNC-3 has not been reported in the VC4 and VC5 neurons [58] that show mis-regulation of IDA-1::GFP in the *pag-3* mutant background. In addition, our rescue experiments demonstrate that PAG-3 acts cell autonomously in VC4 and VC5 with respect to IDA-1 up-regulation. Finally, *unc-3* and *pag-3* mutants have different cell lineage transformations underlying their extra VC and VC-like neuron phenotypes [58]. Taken together, these results suggest that UNC-3 and PAG-3 may act at multiple times in the cell lineage giving rise to these cells, perhaps both together and independently. Characterizing the neurosecretory phenotypes of *unc-3* mutants and exploring the molecular mechanisms that might link UNC-3 and PAG-3 are challenges for future studies.

Given the dramatic up-regulation of *ida-1* gene expression and other DCV-associated gene products in *pag-3* mutants, it is tempting to speculate that PAG-3 directly represses *ida-1* and other DCV component gene expression. We have used the known binding site of PAG-3 *in vitro*, as well as its related mammalian factors, to search bioinformatically for promoters containing matching sequences. Although several of the DCV genes we searched, including *ida-1*, do have sequences within their promoters that resemble the consensus Gfi-1 binding site [59], our attempts (and that of others) to demonstrate direct PAG-3 binding *in vitro* have not been successful. Therefore, the question of whether the effects of *pag-3* mutations on DCV component gene expression are direct or indirect remains unanswered.

Identification of PAG-3 as a factor involved in DCV regulation may have clinical relevance. Recent studies show that mammalian Gfi-1/PAG-3 is involved in the regulation of neuroendocrine cell development and controls neuroendocrine cancer growth [60],[61] (also see review by [62]). Gfi-1 co-expression with neuroendocrine markers, including the DCV-related protein chromogranin A and calcitonin peptide, in small cell lung carcinoma (SCLC) strongly suggests its involvement in the maintenance of the neoplastic phenotype of neuroendocrine lung tumors [60]. We have shown previously that IA-2 is strongly expressed in many small cell lung carcinoma (SCLC) cells with a neuroendocrine phenotype, but not in non-neuroendocrine carcinomas [63]. In fact, IA-2 is expressed in many other, if not all, human neuroendocrine cancer cells examined such as insulinoma [64] and serotonin-secreting tumors of the midgut [65]. Our current results suggest that deregulation of Gfi-1/PAG-3 in neuroendocrine neoplastic disease may severely alter IA-2-related homeostatic control. This dysregulation may contribute directly to the tumor hypersecretion phenotypes that are caused by more than twenty of the different neuropeptides and hormones abnormally released from DCVs such as gastrin-releasing peptide, chromogranin A, atrial natriuretic peptide (ANP), and growth hormone-releasing hormone [66]–[69]. Further studies are required to look for mutations in neuroendocrine pathway genes and to examine directly the role of Gfi-1/PAG-3 and IA-2 in SCLC and related neuroendocrine cancers.

Our current study adds to a growing body of evidence linking IA-2-related protein levels to neuroendocrine secretion and highlights the usefulness of model organisms in dissecting basic biological mechanisms. For human diseases that might benefit from increased neurosecretion, such as diabetes mellitus, targeted knockdown of Gfi-1 function represents a potential therapeutic target. Although there is substantial evidence linking IA-2 protein levels to DCV numbers and secretion, the molecular mechanisms underlying DCV homeostasis and its link to transcriptional control remain unclear. The identification of PAG-3/Gfi-1 as a component of this regulatory system provides another clue for increasing our understanding of these molecular mechanisms.

## Materials and Methods

### Strains

*C. elegans* were grown at 20°C on NGM plates seeded with OP50 bacteria [70] unless otherwise noted. Strains and alleles used were as follows: wild type (N2), CB4856, *ida-1(ok409)*, KM246{gvIs246[*P_ida-1_IA-2::gfp*]}, *pag-3(ls20)*, *pag-3(n3098)*, *pag-3*(*gv560*);gvEx[*pag-3*(+)], *ced-3(n717)*, *unc-3(e151)*, *unc-7(e5)*, *daf-28(sa191)*, *unc-31(e928)*, *unc-64(e246)* and *sta-1(ok587)*. A N2 strain harboring P*_lin-11_*::GFP was kindly provided by Michael R. Koelle. Double mutants were confirmed by phenotype, molecular genotyping of specific alleles, or both.

### Screen for Mutations Leading To Increased IDA-1::GFP Expression

Transgenic L4 larvae of the strain harboring the IDA-1::GFP translational fusion reporter (KM246)[18] were treated with ethyl methanesulfonate (EMS) as described by [70]. Mutagenized worms were placed on seeded 10-cm NGM agar plates and allowed to lay eggs overnight. 2200 individual F1 mutagenized animals were separated onto 550 plates and visually screened three days later as young adults for alterations in IDA-1::GFP in VC and ALA neurons, using a dissecting fluorescence microscopy. Among the mutants examined, we recovered a single penetrant mutation that showed increased IDA-1::GFP expression in neuronal cells. This recessive mutation, *gv560*, was out crossed four times and mapped to LGX.

### Total RNA Preparation and cDNA Analysis

Worms were harvested, washed twice with M9 buffer, and frozen in liquid nitrogen. For RNA preparation, worms were thawed at 65°C for 10 min, and RNA was isolated using the TRIZOL LS Reagent (Invitrogen, Carlsbad, CA). Isolated total RNA was subjected to DNAse treatment and further purified using RNAeasy (Qiagen, Valencia, CA). cDNA was prepared from 5 µg of total RNA in a 100 µl reaction using the SuperScript First-Strand Synthesis System (Invitrogen). The cDNA was used in quantitative real-time PCR (RT-qPCR) using standard conditions. Primer pairs and probes (sequences available upon request) were diluted into 96-well plates at a concentration of 3 µM. Real-time amplification of the cDNA was performed using the TaqMan Universal Master Mix (Applied Biosystems, Foster City, CA). All RT-PCR reactions were carried out and analyzed on an ABI-Prism 9700TH Sequence Detection System (Applied Biosystems), according to the manufacturer's directions. Data were collected using RNA from three independent *C. elegans* populations. To determine the relationship between mRNA abundance and PCR cycle number, all primer sets were calibrated using serial dilutions of cDNA preparations. Relative abundance is reported as the mRNA abundance of each experimental gene relative to the mRNA abundance of several control genes.

### Western Blots and Immunostaining

Western blots were performed using Amersham ECL Plus™ Western Blotting Detection Reagents (GE Healthcare Bio-Sciences Corp, Piscataway, NJ). Immunostaining was performed using a modified protocol [18]. Antisera against an IDA-1 peptide corresponding to amino acid residues 47–61 (CYSSESGSPEPTVLD) was produced in rabbits and purified with Immobilized Protein G Agarose (Pierce Biotechnology, Rockford, IL). Antisera against synaptotagmin (antibody 1095, kindly provided by M. Nonet, Washington University School of Medicine, St. Louis, MO), IDA-1/IA-2 (J. Hutton, University of Colorado at Denver, CO), UNC-31 (K. Miller, Oklahoma Medical Research Foundation, Oklahoma City, OK), and RAB-3 (K. Iwasaki, National Institute of Bioscience and Human Technology, Ibaraki, Japan) were used as described [71]. Antisera against serotonin were from H. Steinbusch (Maastricht University, Maastricht, Netherlands) and detected using the method of Loer (http://home.sandiego.edu/˜cloer/loerlab/anti5htshort.html), anti-synaptobrevin (SNB-1) from Developmental Studies Hybridoma Bank (University of Iowa, IA), and anti-GFP antibodies from Clontech (Palo Alto, CA). Antibodies were detected using rhodamine- or FITC-conjugated goat anti-rabbit or anti-mouse secondary antibodies (Jackson ImmunoResearch, West Grove, PA).

### Analysis of IDA-1::GFP Intensity

Animals were grown at 20°C and different life stages were analyzed from a growing population. For image acquisition, animals were incubated with 10 mM NaN_3_ in M9 buffer for 1 h and mounted on agar pads. Stacks of confocal images with 0.3 to 0.4 µm vertical pitch were recorded with identical exposure times using a Leica TCS SP2 microscope. Maximum intensity projections of all images from a given animal were generated using the NIH ImageJ software package (http://rsbweb.nih.gov/ij/download.html).

### Egg-Laying Assays

Individual young hermaphrodites were placed in 96 wells of a microtiter plate containing 12.9 mM serotonin creatinine sulfate (Sigma) in 100 µl of M9 buffer [72]. After 60 min incubation at room temperature, the eggs laid by each animal were counted. All assays were performed in triplicate (*n* = 10).

### Aldicarb Assays

Chronic effects of aldicarb on mutants were quantified in a growth assay as previously described [18],[39],[73]. Single L1 larva were placed on individual culture plates containing 0, 10, 25, 50, 75, 100, 200, 300, or 400 µM aldicarb and grown at room temperature (22°C) for 96 hr. Growth was then stopped by putting the plates at 4°C. For each aldicarb concentration, the number of progeny was counted as a percentage of the total number of progeny produced on the no-drug control. Triplicate assays were performed and an N2 control set was included with each set of strains to allow for comparisons between different sets of assays.

### Dauer Formation

Adults were allowed to lay eggs on bacterially seeded plates for 3 hr at room temperature and progeny were scored after 48 hr at 25°C as indicated in results [18],[74]. Dauers were distinguished by the distinctive body shape, darkly pigmented intestine, and a constricted pharynx. Each set of assays included all of the relevant strains and the actual ambient incubator temperature surrounding the plates was monitored with a digital thermometer (Barnant Co., Barrington, IL).

### Chromosomal Mapping and Rescue

Genetic mapping was carried out using single nucleotide polymorphisms (SNPs) [75]. The *gv560* mutant allele was crossed into the CB4856 wild type strain [76]. F_2_ Unc progeny with extra VC-like neurons (P9–P12) were scored for the presence of CB4856 SNP markers. Thirteen SNP markers were selected between the interpolated genetic map position 2.86∼23.92 on the X chromosome; pkP6114 (C05C9), pkP6060 (C35C5), pkP6130 (B0198), pkP6125 (C23H4), pkP6131 (K02A4), pkP6115 (C05E7), pkP6161 (F11C1), pkP6132 (F46G10), pkP6133 (C49F8), pkP6164 (R03E1), pkP6116 (F33C8), pkP6087 (C33A11), pkP6093 (F01G12). Detailed location and primer sequence of each SNP are provided at the Genome Sequencing Center (http://genome.wustl.edu/genome/celegans/celegans_snp.cgi). The *gv560* allele was mapped between SNP marker pkP6116 on cosmid F33C8 and pkP6093 on cosmid F01G12. To confirm the gene location of *gv560*, small PCR fragments (1 kb in size) covering the ∼2-kb promoter region, entire coding region, and intron-exon junction regions of *pag-3* were generated for sequencing (ABI 373 DNA sequencer). Primer sequences are available upon request. Further confirmation of the *pag-3(gv560)* mutation was provided by rescuing the mutant phenotypes with a 6.5-kb genomic fragment covering the entire coding region of *pag-3* that was amplified by long-PCR (TaKaRa *LA Taq* DNA Polymerase, Takara Bio USA Corporate, Madison, WI). Animals were injected with the purified PCR products at 1–10 ng/µl together with the co-injection marker pRF4 (*rol-6(su1006)*) at 100 ng/µl and analyzed for rescue of IDA-1::GFP intensity and distribution. To determine whether the effect of *pag-3* mutants on IDA-1::GFP expression is a cell autonomous, the full-length cDNA of *pag-3* was specifically expressed in a limited number of neurons (including VC4 and VC5) driven by the 3-kb promoter of *cat-1* to generate P*_cat-1_*PAG-3 in the PCRII-TOPO vector that was confirmed by sequencing.

### Ultrastructural Analysis

Ten N2, *pag-3(gv560)*, *pag-3(gv560);ida-1(ok409)*, and *ida-1(ok409)* young adult hermaphrodites were subjected to high-pressure freezing. Animals were placed in a 100-µm depth specimen chamber (part no. LZ 02316VN, Technotrade International), frozen rapidly at −176°C under high pressure (pressure >2,100 bar) in a Bal-Tec HPM010 apparatus. Frozen animals were subjected to chemical fixation and dehydration in a Reichart AFS apparatus (Leica): incubation with 0.1% tannic acid and 0.5% glutaraldehyde in anhydrous acetone for 72 h at −90°C followed by several washes in acetone over 6 h. Samples were moved to 2% osmium tetradioxide in anhydrous acetone for 4 h at −90°C before raising the temperature to −20°C at 5°C/h. Samples were then incubated for 16 h at −25°C before raising the temperature to 4°C over 3 h. Samples were then washed in anhydrous acetone for 3 times, each for 15 min and embedded in Epon-Araldite during a 48-h period at 60°C. Blocks were subsequently sectioned and analyzed. Serial sections (80 nm) were counterstained and imaged on CM 120 transmission electron microscope at 120 kV with a Gatan digital camera. A synapse was defined in serial sections that contained the same presynaptic specialization in at least three consecutive sections. Fifteen total ventral cord synapses from two WT animals and seven total ventral cord synapses from two *pag-3(gv560)* animals and eight total ventral cord synapses from two *pag-3(gv560);ida-1(ok409)* or two *ida-1(ok409) animals* were analyzed.
